# Interocular Symmetry in Macular Choroidal Thickness in Children

**DOI:** 10.1155/2014/472391

**Published:** 2014-11-24

**Authors:** Christiane Al-Haddad, Lama El Chaar, Rafic Antonios, Mays El-Dairi, Baha' Noureddin

**Affiliations:** ^1^Department of Ophthalmology, American University of Beirut Medical Center, P.O. Box 110236, Beirut, Lebanon; ^2^Department of Ophthalmology, Duke University Eye Center, NC, USA

## Abstract

*Objective.* To report interocular differences in choroidal thickness in children using spectral domain optical coherence tomography (SD-OCT) and correlate findings with biometric data. *Methods.* This observational cross-sectional study included 91 (182 eyes) healthy children aged 6 to 17 years with no ocular abnormality except refractive error. After a comprehensive eye exam and axial length measurement, high definition macular scans were performed using SD-OCT. Two observers manually measured the choroidal thickness at the foveal center and at 1500 *µ*m nasally, temporally, inferiorly, and superiorly. Interocular differences were computed; correlations with age, gender, refractive error, and axial length were performed. *Results.* Mean age was 10.40 ± 3.17 years; mean axial length and refractive error values were similar between fellow eyes. There was excellent correlation between the two observers' measurements. No significant interocular differences were observed at any location. There was only a trend for right eyes to have higher values in all thicknesses, except the superior thickness. Most of the choroidal thickness measurements correlated positively with spherical equivalent but not with axial length, age, or gender. *Conclusion.* Choroidal thickness measurements in children as performed using SD-OCT revealed a high level of interobserver agreement and consistent interocular symmetry. Values correlated positively with spherical equivalent refraction.

## 1. Introduction

The use of imaging to assess the choroidal layer is valuable in the diagnosis and monitoring of several ocular conditions such as central serous retinopathy [1, 2], polypoidal choroid vasculopathy [3], and high myopia [4, 5]; abnormal choroidal thickness has been implicated in the pathophysiology of those conditions. Among the many modalities (high-frequency ultrasound and magnetic resonance imaging) [6, 7] used to image the choroid, optical coherence tomography (OCT), especially spectral domain-OCT (SD-OCT) devices, is increasingly being used for that purpose due to ease of use and higher reproducibility. Newer imaging techniques like enhanced depth imaging (EDI) [8] and averaging of high-density line scans [2] have improved visualization of the chorio-scleral interface.

Imaging can be especially helpful in diagnosing diseases that have asymmetrical or unilateral presentations, early in their course. However, some degree of nonpathologic asymmetry can exist between fellow eyes as demonstrated by previous studies on retinal nerve fiber layer and macular parameters [9–11]. Recently a number of papers discussing choroidal thickness have emerged comparing normal values across different age groups, in addition to studies on choroidal changes in children with amblyopia [12–14]. It is important to define the limits of normal interocular variations in choroidal thickness in order to differentiate pathologic from nonpathologic asymmetry. Two studies have reported interocular differences in choroidal thickness in adult populations; neither showing any statistically significant differences [8, 15]. In children, the limits of nonpathologic interocular asymmetry have not been explored although choroidal thickness measurements have been reported to be influenced by age [3, 10, 16].

In our cross-sectional study, we used the Cirrus SD-OCT to image healthy children between 6 and 17 years of age to determine whether any significant nonpathologic interocular variation in choroidal thickness exists. We also correlated measurements with biometric data.

## 2. Methods

### 2.1. Study Population

This was a cross-sectional study of healthy white and Middle Eastern children 6 to 17 years of age visiting the pediatric ophthalmology clinic at the American University of Beirut Medical Center for refractive error evaluation over a year. This study was approved by the American University of Beirut Institutional Review Board. Written parental informed consent was obtained from parents or legal guardians; children and adolescent assent forms were also provided for children above 7 years of age. Detailed demographic data were obtained during the clinic encounter. Included in the study were subjects with no ocular abnormality except refractive error less than 7.00 diopters (of hyperopic or myopic spherical equivalent) and normal fundoscopy. Excluded were patients with history of intraocular surgery, strabismus, anisometropia more than 1.50 diopters, amblyopia, retinal pathology, glaucoma, high intraocular pressure, optic nerve cup to disc ratio > 0.5, or asymmetry of > 0.2 between fellow eyes. Patients with history of prematurity, neurologic, metabolic, or other systemic diseases were also excluded.

### 2.2. Ocular Examination

All subjects received a comprehensive ophthalmologic exam by a pediatric ophthalmologist (Christiane Al-Haddad). The visual acuity of each eye was recorded using the Snellen chart; intraocular pressure assessment, motility exam, stereoacuity testing, slit lamp exam, cycloplegic refraction, and dilated fundoscopy were performed. Pupils were dilated using phenylephrine 2.5% and cyclopentolate 1% eyedrops. Manual retinoscopy was performed 30 minutes after the last drop by a pediatric ophthalmologist (Christiane Al-Haddad). This was also confirmed by automated refraction (Canon RK-F1 autorefractor; Canon, Tokyo, Japan). Axial length (AL) measurements were obtained using the IOL master (Carl Zeiss AG, Oberkochen, Germany). Multiple AL measurements (at least 3) were taken and an average value was recorded.

### 2.3. Spectral Domain OCT Imaging

Cirrus HD-OCT (Carl Zeiss, Dublin, CA, USA) device was used to obtain high-definition images. Signal strength of 6 or more was considered acceptable. The right eye was scanned first followed by the left eye. Internal fixation was used to ensure proper alignment of the eye and the best centration on the foveal center. All imaging was performed by an experienced ophthalmic photographer or one of the authors. SD-OCT images through the fovea were obtained for each patient using an internal fixation target to measure choroidal thickness and perform initial comparisons between fellow eyes. Both horizontal and vertical single line scans were performed first for the right eye then the left eye. Multiple scans were performed and those with the best definition of the sclerochoroidal interface and centration on the fovea (as marked by the bright foveal reflex) were used for analysis. Individual thicknesses were measured manually using the calipers available on the SD-OCT machine by two separate masked observers. Subfoveal choroidal thickness was measured as the perpendicular distance from the outer aspect of the retinal pigment epithelium (RPE) to the inner aspect of the sclera. In most of the images there was a hyperreflective line between the choroid and the sclera marking the sclerochoroidal interface. When the boundary was unclear in the foveal center, a smooth line joining the outer limits of the choroid vascular space was drawn to delineate the scleral layer; the choroidal thickness would then be measured up to that limit. Choroidal thickness was similarly measured at 1500 *µ*m nasally and temporally from the foveal center in horizontal scans and at 1500 *µ*m superiorly and inferiorly from the foveal center in vertical scans.

### 2.4. Statistical Analysis

Data were entered in a Microsoft Excel sheet and then transferred to the Statistical Package for Social Sciences program (SPSS, version 19) for data management and analyses. Descriptive statistics were reported as mean and standard deviation, as well as the 5th and 95th percentiles for continuous variables, and the number and percent for categorical ones. Mean interocular differences for choroidal thicknesses were calculated by subtracting left eye parameters from right eye parameters, and their respective *P* values were reported using the paired sample *t*-test. All studied measurements had a normal distribution as demonstrated by the kurtosis test. Mean differences were thus positive when right eyes had higher values and negative when left eyes had greater measurements. Subjects were also divided into two age groups, those younger than 11 years (6–10 years) and those 11 years and older (11–17 years), to explore any differences related to growth. Correlation analyses were utilized to assess the effect of demographic characteristics (age, gender, spherical equivalent, and axial length) on the different thicknesses, where the correlation coefficient was reported along with the *P* value. Multiple regression analysis was also performed and results were consistent with the correlation analysis. *P* value <0.05 was considered to indicate statistical significance. We also computed the intraclass correlation coefficients (ICCs) to measure agreement between the two observers. For each of the studied thicknesses, an average of the two observers' measurements was computed and used for analysis. In addition, Bonferroni correction was utilized when comparing choroidal thicknesses in right and left eyes because several statistical tests were performed on the same data set.

Scatter plots were used to explore the data and to analyze agreement among measurements between fellow eyes of the same subject. Bland-Altman plots for interocular choroidal thickness agreement were performed by calculating the difference between right and left eyes plotted against the mean thickness of the two eyes. Horizontal dashed lines were drawn at the 95% limits of agreement, which were defined as the mean difference ±1.96 × standard deviations of the differences.

## 3. Results

A total of 113 children were enrolled in the study. Twenty-two subjects were later excluded: 5 had decentered scans and 17 did not have a clear view of the choroidal layer boundaries. The final analysis included 91 subjects (182 eyes), 56 females and 35 males, with mean age of 10.40 ± 3.17 years. Baseline demographic characteristics are reported in Table 1. No significant difference was noted when comparing spherical equivalent and axial length between right and left eyes.

Choroidal thickness measurements were taken by two observers. Interobserver correlation was high with intraclass correlation coefficient (ICC) values higher than 0.9 for all parameters studied (Table 2). Mean choroidal thicknesses were computed by averaging the two observers' measurements. The central foveal thickness measured 318.38 ± 53.55 *μ*m in right eyes and 311.59 ± 50.72 *μ*m in left eyes (Table 3). Mean values of the choroidal thicknesses were calculated, as well as the median, 5th percentile, and 95th percentiles (Table 3).

Interocular comparisons for the different thicknesses revealed no significant differences (*P* > 0.05). Bland-Altman plots also showed the differences to be scattered well within the limits of agreement between right and left eyes. This also applied when subjects were subgrouped into the younger (6–10 years, *n* = 49) and the older (11–17 years, *n* = 42) age groups. The younger group consisted of 18 males and 31 females, while the older group included 17 males and 25 females, which was similar to the gender distribution in the group as a whole. When comparing choroidal thickness in different locations, the trend was for the central thickness to be highest followed by the inferior thickness, temporal thickness, superior thickness, and finally nasal thickness, but this was not statistically significant (Tables 3 and 4). This trend was also consistent in the two age subgroups. The younger age group tended to have thicker choroids at all locations, but this was not statistically significant (Table 4). Mean and median interocular differences were calculated for all choroidal thicknesses. There was a trend for right eyes to have higher values in all thicknesses, except the superior thickness where the left eyes had the higher values both in all subjects and in the different age subgroups (Table 5).

Correlation analysis was performed between the measured choroidal thicknesses and four parameters: gender, age, axial length, and spherical equivalent (Table 6). Multiple regression analysis results were consistent with the correlation analysis. Most of the choroidal thickness values correlated positively with spherical equivalent and this was statistically significant. There was a trend for choroidal thickness to be negatively correlated with female gender, increasing age, and increasing axial length. This, however, did not reach statistical significance. It is noteworthy to mention that the patient baseline parameters were significantly correlated as expected: age being positively correlated with axial length (*P* = 0.04) but negatively correlated with spherical equivalent (*P* = 0.003) and axial length being negatively correlated with spherical equivalent (*P* = 0.01).

## 4. Discussion

This study aimed at examining interocular symmetry of macular choroidal thickness as measured by Cirrus SD-OCT in children between 6 and 17 years of age. We did not detect statistically significant interocular differences at any of the studied locations (subfoveal, nasal, temporal, superior, and inferior); this was also consistent when analyzing the younger and older subgroups separately. When comparing the different thicknesses, there was a trend for the central subfoveal thickness to be highest followed by the inferior, temporal, superior, and finally nasal thickness. Measurements correlated positively with spherical equivalent.

A few previous studies in the literature have documented the presence of nonpathologic interocular differences in OCT parameters in children < 16 years [9–11]. These reports mainly studied retinal nerve fiber layer and macular parameters showing consistent significant differences. With the introduction of new OCT upgrades, the choroidal layer is now better visualized and although there is no commercially available software to measure it, manual measurements are feasible with the high definition images obtained using SD-OCT. Several reports in the literature have shown that choroidal measurements are reproducible in children and many have reported normal choroidal thickness values in healthy children at different locations [16–21]. These pediatric studies either used randomly one eye for analysis or grouped right and left eyes together. Bidaut-Garnier et al. reported a high correlation in the choroidal thickness measurements performed between fellow eyes [21]. The current study is the first to explore interocular differences in choroidal thickness in children.

Interobserver agreement in choroidal thickness measurements was high in our study with ICC above 0.9 for all locations measured (Table 2); this is in agreement with the similar study by Chen et al. [15]; Cho et al. also recorded high interobserver ICCs in a repeatability study of choroidal thickness OCT measurements; however, repeatability was lower in cases with thicker choroids [22]. Although choroidal thickness has to be measured manually, the high definition images provided by SD-OCT do provide clear boundaries for the layer in question; using gray scale images and enhancing the contrast additionally facilitates measurements. Standard deviations (SDs) and ranges for the different thicknesses were in agreement with other authors' works [16–20]. Our SDs for the mean values ranged between 50 and 63 *μ*m; other authors reported SDs ranging between 60 and 85 *μ*m [18–21, 23]. Even higher SD values were recorded when measuring choroidal thicknesses in adults, reaching up to 103 *μ*m [15].

Interocular symmetry in choroidal thickness has been studied in one report in adults [15]; no similar report is available in the pediatric population. Chen et al. found no significant differences between right and left eyes in 50 adult subjects, with a median (range) interocular difference (right minus left eye) in foveal choroidal thickness of 21 (0.38, 135.13) *μ*m and mean (SD) difference of 1.0 (42) *μ*m [15]. Spaide et al., in a feasibility study of choroidal thickness measurements on OCT, described a high correlation between right and left eyes in 17 adult subjects with an absolute mean interocular difference of 17 *μ*m [8]. In our study, we computed interocular differences in choroidal thicknesses in 91 healthy children; median (range) difference for the subfoveal thickness was 9 (−87, 117) *μ*m and mean (SD) was 6.79 (43.63) *μ*m. Although the interocular difference was small, the range between minimum and maximum values was large (Tables 4 and 5); this was similarly the case in the work by Chen et al. where ranges were even wider [15]. This could be attributed to the subjective way of measurement. No alternative method exists currently to obtain more objective measurements. Choroidal thickness is also known to vary by age and diurnal rhythm, which could contribute to the wide range of values observed. Although differences were nonsignificant in our population, right eyes tended to have higher values for all thicknesses except the superior value. This applied for both the younger and older age groups. Chen et al. also found a trend for higher values in right eyes except in the temporal thickness [15]. We have no explanation for this trend, but studies on RNFL and macular thickness symmetry have demonstrated significantly thicker nasal, temporal, and inferior RNFL quadrants in right eyes and thicker outer nasal and outer inferior macular quadrants in left eyes [9].

Comparing the choroidal thicknesses in the various locations studied, no significant difference was noted. There was a trend for subfoveal thickness to be highest, followed by the inferior, temporal, superior, and nasal thickness. This was observed both in right and in left eyes and in both the younger and older age groups. Chen et al. similarly measured choroidal thickness at different locations but at 3 mm from the fovea [15], while in the current study a distance of 1500 *μ*m was chosen. This distance was selected in conformity with our earlier study on interocular symmetry in retinal parameters [9]. The choroidal thickness profile described by Chen et al. was slightly different from ours with the foveal thickness being highest, followed by the superior, inferior, temporal, and then nasal thickness [15]. Again no interocular differences were encountered. There is no consensus in the literature on a macular choroidal thickness pattern, but pediatric studies have agreed that the thinnest measurements were obtained nasally [16, 17, 20, 24, 25] and inferonasally [17, 19]. Measurements in other locations did not show the same consistency.

In the correlation analysis, only spherical equivalent had a significant positive correlation with choroidal thickness in our study (Table 6). Read et al. demonstrated that the choroidal layer was thinner (on average 50 *μ*m) at all locations in myopic children when compared to nonmyopes [17]. Others have observed varying correlations with age [17, 19, 23], axial length [16, 18, 19], and body weight [16, 17]. Although we observed a negative correlation with age and axial length, it did not reach statistical significance. Fujiwara et al. and Ruiz-Moreno et al. have shown that children less than 10 years of age exhibited thicker choroids than older children [4, 20]. This was the rationale for the cut-off at 10 years in our age subgroups. We did not however notice any discrepancies between the 2 age groups with respect to interocular differences or thickness profiles. The relationship between choroidal thickness and age is not a simple one in children. Central choroidal thickness was remarkably higher in newborns as compared to adults [25]. Read et al. however showed that in early years (4–6 years) the choroid was thinner than in later years in childhood; then it rapidly increased to plateau in early adolescence [19]. They attributed that to the natural growth of the eye [19]. More recent papers have shown variable correlations with age with some showing negative correlations and others none [12, 13, 26].

Chen et al. described a weak correlation with axial length only in subjects with increased axial length measurements; refractive error data was not available [15]. Although spherical equivalent and axial length are closely related, they are not equivalent. Our subjects belonged to a group of mostly uniform axial length (Table 1) and we purposely excluded children with high refractive errors to avoid any outliers. Disparities in correlations of choroidal thickness with axial length among different studies could be attributed to the use of different OCT devices, varying axial length distributions in subjects, and varying distances from the foveal center where paramacular choroidal thickness measurements were performed.

Limitations of this study include the relatively small sample size and the uniform ethnic group (white Middle Eastern). We also did not account for the possible diurnal variation in choroidal thickness; our measurements were taken at various times during the day. Measurements were performed manually; currently no commercial software exists to accomplish this more objectively. However, two masked observers undertook the measurements and ICC values were high. Another limitation is the fact that 22 subjects could not be included in the study because the image quality made it difficult to perform measurements. Thicker choroids may have caused this, which might bias the results towards subjects with thinner choroids.

In conclusion, foveal choroidal thickness measurements in children obtained using SD-OCT were reliable and showed consistent interocular symmetry at all locations studied. Values demonstrated a positive correlation with spherical equivalent. This information is valuable both to the clinician while interpreting any observed asymmetry in the choroidal layer on OCT scans and to the researcher while selecting one eye in studies using OCT.

## Figures and Tables

**Table 1:** Demographic characteristics of the study population.

Total number of patients (eyes)	91 (182)
Mean age (years) ± SD	10.40 ± 3.17
Gender	
Females	
Number	56
Mean age (years) ± SD	10.20 ± 3.42
Males	
Number	35
Mean age (years) ± SD	10.52 ± 3.03
Mean spherical equivalent (D)	
Right eyes ± SD	0.11 ± 1.83
Left eyes ± SD	0.16 ± 1.77
Mean axial length (mm)	
Right eyes ± SD	23.45 ± 1.05
Left eyes ± SD	23.43 ± 1.07

**Table 2:** Mean choroidal thickness values as reported by the two raters and their intraclass correlation coefficients (ICCs).

	Reader 1	Reader 2	ICC
Choroidal thickness (*μ*m)			
Central			
OD	318.84 ± 52.99	318.22 ± 53.86	0.99
OS	309.03 ± 54.39	310.60 ± 53.19	0.97
Superior			
OD	296.80 ± 55.95	293.37 ± 54.63	0.97
OS	300.54 ± 53.02	297.60 ± 53.72	0.97
Nasal			
OD	270.70 ± 61.32	262.42 ± 67.16	0.96
OS	261.48 ± 58.75	254.46 ± 64.41	0.94
Temporal			
OD	311.58 ± 50.09	305.47 ± 54.12	0.95
OS	298.00 ± 60.69	296.25 ± 62.42	0.99
Inferior			
OD	306.75 ± 51.81	302.86 ± 53.03	0.97
OS	302.74 ± 54.82	299.34 ± 56.99	0.95

**Table 3:** Choroidal thickness values in right and left eyes.

	Mean ± SD^*^	Median (min, max)	5th percentile	95th percentile	*P* value	Bonferroni *P* ^∧^
Choroidal thickness (*μ*m)						
Central					**0.14**	**0.03**
OD	318.38 ± 53.55	326 (247, 426)	221.20	404.80		
OS	311.59 ± 50.72	313 (187, 422)	215.60	394.20		
Superior					**0.24**	**0.05**
OD	294.99 ± 54.98	300 (142, 463)	207.20	386.80		
OS	299.07 ± 52.84	298 (172, 404)	208.80	376.00		
Nasal					**0.14**	**0.03**
OD	266.64 ± 63.49	268 (137, 445)	159.00	366.80		
OS	259.91 ± 57.24	263 (126, 423)	156.90	344.40		
Temporal					**0.06**	**0.02**
OD	308.76 ± 51.23	313 (127, 424)	219.80	386.20		
OS	299.49 ± 57.21	297 (138, 416)	176.60	396.40		
Inferior					**0.64**	**0.13**
OD	305.07 ± 51.95	309 (174, 405)	209.20	389.80		
OS	302.72 ± 52.52	311 (166, 414)	199.00	383.40		

^*^Mean choroidal thicknesses were computed by averaging the two observers' measurements.

^∧^Bonferroni correction for *P* < 0.01 is significant.

**Table 4:** Comparison of choroidal thicknesses by age groups.

	6–10 years			11–17 years		
	Mean ± SD	Median (min, max)	*P* value	Mean ± SD	Median (min, max)	*P* value
Choroidal thickness (*μ*m)						
Central			**0.11**			**0.63**
OD	321.95 ± 53.11	316 (208, 426)		314.21 ± 54.41	331 (179, 396)	
OS	312.39 ± 53.60	311 (187, 422)		310.67 ± 47.76	316 (197, 397)	
Superior			**0.27**			**0.59**
OD	299.99 ± 57.70	296 (170, 463)		289.15 ± 51.69	301 (142, 395)	
OS	306.84 ± 50.53	315 (173, 404)		292.96 ± 52.16	289 (172, 392)	
Nasal			**0.24**			**0.36**
OD	267.45 ± 66.64	268 (137, 445)		265.70 ± 60.41	272 (154, 374)	
OS	260.42 ± 64.18	269 (126, 423)		259.32 ± 48.67	260 (156, 370)	
Temporal			**0.48**			**0.07**
OD	312.13 ± 47.90	316 (221, 424)		304.82 ± 55.18	312 (127, 385)	
OS	308.19 ± 44.27	302 (224, 414)		289.33 ± 68.51	286 (138, 416)	
Inferior			**0.99**			**0.47**
OD	307.11 ± 50.29	314 (184, 404)		302.68 ± 54.33	314 (174, 405)	
OS	307.09 ± 49.25	300 (166, 414)		297.62 ± 56.25	300 (166, 414)	

**Table 5:** Interocular differences in choroidal thicknesses.

	All ages		6–10 years		11–17 years	
	Mean ± SD	Median (min, max)	Mean ± SD	Median (min, max)	Mean ± SD	Median (min, max)
Choroidal thickness (*μ*m)						
Central	6.79 ± 43.63	9 (−87, 117)	9.57 ± 40.83	12 (−87, 117)	3.54 ± 46.97	2 (−87, 96)
Superior	−5.45 ± 43.91	−1 (−99, 134)	−6.88 ± 43.39	−13 (−99, 134)	−3.81 ± 45.04	9 (−78,78)
Nasal	6.73 ± 42.85	3 (−232, 256)	7.00 ± 41.61	4 (−68, 107)	6.40 ± 44.78	−3 (96, 133)
Temporal	9.27 ± 46.09	−3 (−74, 153)	3.98 ± 39.03	−3 (−71, 93)	15.52 ± 52.98	−1 (−74, 153)
Inferior	2.35 ± 48.33	3 (−131, 137)	0.00 ± 51.71	4 (−131, 137)	5.07 ± 44.54	2 (−74, 127)

**Table 6:** Pearson correlation coefficients between choroidal thicknesses and age, gender, axial length (AL), and spherical equivalence.

	Right eyes					Left eyes				
	Central	Superior	Nasal	Temporal	Inferior	Central	Superior	Nasal	Temporal	Inferior
Female versus male	0.05	−0.10	−0.08	−0.17	−0.06	−0.09	−0.10	−0.02	0.05	0.01
Age (years)	−0.02	−0.12	0.04	−0.05	−0.02	0.01	−0.14	−0.01	−0.09	−0.08
AL (mm)	−0.10	−0.15	−0.04	−0.03	−0.08	−0.17	−0.17	−0.06	−0.09	−0.16
Spherical equivalence (D)	0.23^*^	0.19	0.21^*^	0.22^*^	0.08	0.28^*^	0.33^*^	0.27^*^	0.22^*^	0.28^*^

^*^Correlation is significant (*P* < 0.05).
